# Society and the Microbiome: A Biopsychosocial Window Into Comprehensive Well‐Being: A Review

**DOI:** 10.1002/hsr2.72162

**Published:** 2026-05-04

**Authors:** Jing Li, Yang Yang, Yufeng Wang, Ji'An Liu, Xufeng Huang, Zhengrui Li, Ju Li

**Affiliations:** ^1^ Shanghai Stomatological Hospital & School of Stomatology Fudan University Shanghai China; ^2^ Heilongjiang University of Traditional Chinese Medicine Harbin China; ^3^ Yunnan Key Laboratory of Dai and Yi Medicines, YunnanUniversity of Chinese Medicine, College of Basic Medical Science Yunnan University of Chinese Medicine Kunming China; ^4^ Department of Oral and Maxillofacial‐Head and Neck Oncology, Shanghai Ninth People's Hospital Shanghai Jiao Tong University School of Medicine; College of Stomatology, Shanghai Jiao Tong University; National Center for Stomatology; National Clinical Research Center for Oral Diseases; Shanghai Key Laboratory of Stomatology; Shanghai Research Institute of Stomatology Shanghai China; ^5^ Faculty of Dentistry University of Debrecen Debrecen Hungary; ^6^ Department of Oral and Cranio‐Maxillofacial Surgery, Shanghai Ninth People's Hospital Shanghai Jiao Tong University School of Medicine; College of Stomatology, Shanghai Jiao Tong University; National Center for Stomatology; National Clinical Research Center for Oral Diseases; Shanghai Key Laboratory of Stomatology Shanghai China

**Keywords:** gut microbiota, human microbiota, social behavior, societal dynamics

## Abstract

**Background and Objectives:**

In addition to biological factors, human social behavior, societal structures, and environmental contexts significantly influence the human microbiome. This review examines how socially relevant factors relate to the microbiome to clarify underlying mechanisms and health impacts, aiming to inform effective preventive and therapeutic strategies.

**Methods:**

We synthesized relevant literature from PubMed using a biopsychosocial framework, integrating structural socio‐political and contextual factors to elucidate interactions between social behavior and the microbiota.

**Results and Conclusions:**

Social behavior shapes the microbiome through complex biological, psychological, and socio‑cultural pathways, with health consequences involving immune, mental, and metabolic functions. Future research should clarify the fundamental drivers of this relationship, identify individual differences, and employ longitudinal designs to measure sustained effects.

## 1 Introduction

The human microbiome plays an instrumental role in sustaining health, comprising a diverse assembly of bacteria, viruses, fungi, and protozoa across bodily locales [1, 2, 3, 4, 5]. Comprising a diverse assembly of bacteria, viruses, fungi, and protozoa, the human microbiome predominantly resides in locales such as the gut, skin, oral cavity, and respiratory tract [4]. Over recent decades, seminal works have delineated the pivotal functions of the human microbiome in processes like digestion [6], immune regulation [7], nutrient absorption [8], and mental health [9]. Additionally, a substantial corpus of evidence suggests that microbiome imbalances, termed dysbiosis, might be intrinsically linked to the genesis and progression of an array of pathologies [10, 11, 12, 13, 14, 15], including obesity, cancers, diabetes, cardiovascular diseases, autoimmune disorders, and mental illnesses. Beyond biology, the microbiome is also shaped by societal structures, socio‐political factors, and environmental contexts. Urbanization, industrialization, and socioeconomic inequalities have a profound influence on microbiome diversity. For example, individuals in urbanized settings often exhibit reduced microbial diversity due to increased exposure to pollutants, processed foods, and antibiotics. Policies and practices governing food production, sanitation, and antibiotic use further impact microbial health at a population level.

In recent years, an increasing number of researchers have begun investigating human social behavior's impact on the microbiota [16, 17, 18]. Social behavior encompasses various aspects [19, 20], such as family structure, intimate relationships, friendships, and workplace relationships, which may affect an individual's microbiota through different mechanisms [17, 21, 22, 23]. For instance, intimate behaviors among family members can facilitate the transmission and sharing of microbes [24]. Members of the same household may exhibit similar microbiota compositions [6], potentially due to shared living environments, dietary habits, and lifestyles. Moreover, individuals within social networks may influence one another's microbiotas through interactions and behavioral patterns [17, 25]. Social behavior, while a critical component of this dynamic, operates within a structural framework that includes access to healthcare, environmental conditions, and cultural norms. For instance, socioeconomically disadvantaged populations may experience greater exposure to environmental stressors, limited access to fresh produce, and higher consumption of processed foods, all of which can negatively impact microbiome diversity and health (Zhu et al., 2023). Moreover, public health policies, housing density, and urban planning influence how microbial transmission occurs within communities.

**FIGURE 1:**
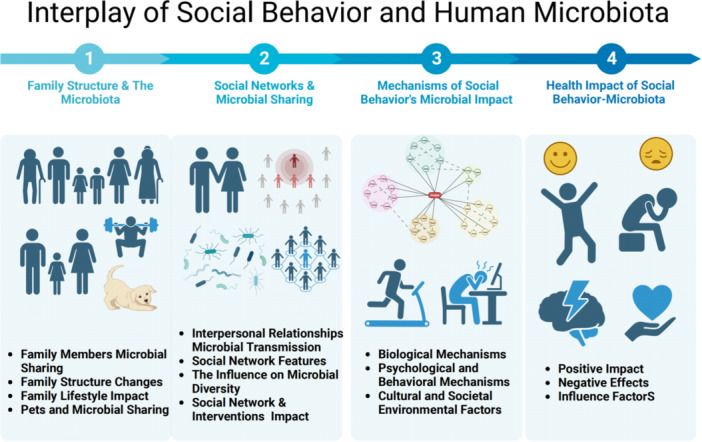
The intricate interplay between human social behavior and the microbiota.

This review employs a biopsychosocial framework to examine the interplay between social behaviors and microbiota, integrating structural socio‐political and contextual factors (Figure 1). To support this analysis, we conducted a literature search in PubMed using combinations of keywords, including “social behavior,” “microbiome,” “microbiota,” “psychological stress,” and “mechanisms.” To ensure timeliness, the search was limited to original research or review articles published in English within the last decade. Understanding these interactions can reveal pathways through which societal structures shape microbial ecosystems, offering insights for policy makers, healthcare providers, and public health advocates.

## 2 Family Dynamics and Microbial Interactions

### 2.1 Familial Microbiota Symbiosis

The exchange of microbes within family circles is a crucial area for scholarly exploration. Due to similarities in environmental exposure, behavioral patterns, and living conditions, family members often display notable similarities in their microbial communities [26, 27]. Research highlights this congruence in gut, oral, and skin microbiomes, especially pronounced among genetically related individuals compared to those unrelated or not cohabiting [28]. Advanced genomic sequencing has revealed significant similarities in antibiotic resistance gene profiles within these familial groups, suggesting common vulnerabilities or resistances to specific health conditions [27].

The influence of kinship on microbial alignment is particularly striking. Newborns and their mothers, for example, show a substantial similarity in gut microbiota [29], likely due to microbial transmission during childbirth and breastfeeding. Delivery mode fundamentally determines early‐life microbiome acquisition. Vaginally delivered infants acquire communities resembling maternal vaginal microbiota, while cesarean‐delivered infants harbor skin‐like communities [30], a difference that may persist. At 3 months, cesarean‐delivered infants have lower beneficial Bifidobacterium and Bacteroides (both *p* < 0.05), more potential pathogens, and compositional differences lasting to 12 months [31], potentially increasing disease risk. Feeding mode also matters: formula‐fed infants show higher diversity than breastfed infants [32]. These findings have clinical implications. Clarifying delivery and feeding practices, understanding their interactive effects, using microbial biomarkers, and developing targeted therapies may improve immune‐related disease management. Siblings also show microbial concordance, likely from shared environments, habits, and genetics.

Siblings also demonstrate increased microbial alignment, likely due to shared living environments, lifestyle habits, and genetic factors. This leads to the hypothesis that each family develops its unique microbial lineage, a concept we term the “Familial Microbiota Paradigm.” It has also been demonstrated that family members, including people other than mothers, can pass on bacteria to their offspring [33]. This opens up potential strategies for shaping childhood microbiome using this “familial transmissible bacteria,” which is measurable across children and their families.

### 2.2 Microbial Dynamics in Changing Family Structures

Family structure shifts significantly affect individual microbiomes [34, 35]. Major life events like birth, marriage, or divorce bring changes in daily routines and living environments, leading to alterations in microbial communities. For instance, a newborn's microbiome undergoes significant changes after birth, transitioning from a primarily maternal to a more diverse external microbial environment. This initial microbial development in newborns is predominantly influenced by maternal microbiota and evolves under the influence of external factors. Similarly, the act of marrying or moving in with a partner can increase microbial sharing due to shared living spaces and synchronized lifestyles. Conversely, the end of a relationship might reduce this microbial sharing. In fact, research indicates that cohabiting couples, particularly those with deep relationships, have a more diverse microbiome than single people. As stress has been linked to a decrease in microbial diversity [17, 36], interpersonal stress, which can occur during social contact and lead to interpersonal conflict, the breakdown of love relationships, and so forth, can impact the microbiome. Moreover, research has shown a correlation between depression and a reduction in the richness of the gut microbiota [37]. It is hypothesized that romantic relationship disintegration may be connected to the following since it is commonly linked to living alone, experiencing stress, and having a depressed disposition [38]. Emerging evidence has begun to elucidate the underlying mechanisms linking social dynamics to microbial and health outcomes. A recent animal study demonstrated that social connection enhances gut microbiota diversity and stabilizes its composition, and can further feedback to regulate social behavior [39]. This bidirectional regulatory effect provides additional evidence for the close link between the microbiota and social behavior, which is mediated by specific bacterial species such as *Enterococcus faecalis* that inhibit excessive activation of the HPA axis, thereby downregulating neuronal activity in relevant brain regions [40]. Notably, the efficacy of microbiome‐based interventions for psychiatric conditions such as depression and social isolation has been demonstrated in both animal models and clinical studies.

### 2.3 Influence of Family Lifestyle on Microbiota

Family dietary habits, hygiene practices, sleep habits, and physical activity levels significantly impact the microbiomes of family members [35]. Dietary preferences are particularly influential in shaping the gut microbiome. Prolonged consumption of a nutritious diet has been linked to elevated α‐diversity of microbial gene families and metabolic pathways, as well as modified symbiotic roles that are pertinent to human nutrition and well‐being [41]. Families may share similar hygiene practices and use the same hygiene products, which can impact the skin microbiomes and affect skin health [42, 43].

Exercise routines may indirectly affect microbiome balance and can increase the diversity of the microflora, improve the Bacteroidetes–Firmicutes ratio, which may help lower body weight, obesity‐related pathologies, and gastrointestinal disorders. Exercise has also been shown to promote the growth of beneficial microbes that enhance mucosal immunity, potentially reducing the risk of metabolic diseases such as obesity, and to support bacteria that produce compounds protective against gastrointestinal disorders [44].

Further addictive habits, including smoking, alcohol, drugs, as well as other minor addictive substances, can also impact microbiome [45, 46, 47]. The microbiome, in particular, has been implicated in opioid‐seeking behavior [48]. These hypotheses, however, require further empirical validation. A recent cluster randomized controlled trial demonstrated that lifestyle interventions targeting community residents positively influenced the cultivation and maintenance of healthy lifestyle behaviors. These changes were also associated with alterations in the gut microbiota, providing strong evidence that behavioral patterns shape the microbiome. This highlights the importance of establishing healthy lifestyles within small social units, which may involve mechanisms such as behavioral imitation and microbial exchange [49].

### 2.4 Companion Animals and Microbiome Interactions

Pets significantly contribute to the microbial dynamics in households [50]. Families with pets often show increased microbial sharing with their animal companions [51, 52]. This interaction has dual effects. On the one hand, pets can increase human microbial diversity and promote health. For example, in families with infants, pet ownership has been shown to increase the abundance of bacteria negatively associated with childhood obesity and allergic diseases [53]. Prenatal and early‐life exposure to pets, particularly dogs, is also associated with beneficial changes in the infant gut microbiome [54, 55].

On the other hand, pet exposure may introduce potential pathogens, potentially increasing health risks [51]. For instance, women living with pet dogs have been reported to harbor increased levels of pathogenic *E. coli* associated with urinary tract infections [56]. More notably, maternal prenatal dog exposure has been positively associated with ADHD in male offspring [57].

Therefore, the differential effects of pets on various diseases and populations require further investigation. Future research should also explore the specific contributions of different pet types to microbiome alterations.

## 3 Social Connectivity and Microbial Commensalism

### 3.1 Interpersonal Dynamics and Microbial Transfer

Social networks, intricate mosaics of human interactions and relationships, significantly influence microbial exchange [58, 59]. Physical interactions, such as embraces, intimate kisses, or even handshakes, serve as conduits for microbial transmission. Empirical studies have shown that close interactions, like deep kissing, result in notable convergence of oral microbiota, with the degree of convergence intensifying with longer contact duration. Additionally, shared use of everyday items or communal spaces, from utensils to office desk and equipment, can foster microbial sharing among individuals. Microbe transfer between an inhabitant and the office environment depends on a variety of parameters, some of which are still unknown [60]. How built environments and human activities alter the indoor microbiome remains a fundamental question for future exploration.

The microbial transfer dynamics also depend upon their inhabitant site. During infancy, there is significant and consistent transfer of the gut microbiome from mother to child which persists for subsequent ages. On the other hand, the oral microbiome spreads primarily through horizontal transmission, with cohabitation duration playing a key role. Significant strain sharing has been observed among cohabiting individuals, with cohabitation exerting a greater impact on strain sharing than genetics or age [61]. Thus long‐term close contact between people transfers strains of microorganisms, which shapes the genetic composition of the microbiome and, consequently, the capacity for host‐microorganism interaction and metabolism.

### 3.2 Structural Characteristics of Social Networks

Family structure encompasses the composition, stability, and relational patterns of an individual's primary living environment. This includes both relatively stable configurations, such as different forms of family composition, and dynamic transitions driven by significant life events. The inherent structure of social networks, including their density, layout, and relational proximity, plays a pivotal role in microbial exchange [58, 62].

Nevertheless, little is known about how social interactions beyond household contacts and cohabitants affect the human microbiome. However, the influence of an individual's wider social network on their microbiome composition is undoubtedly significant. In addition, the type and frequency of social interactions such as handshakes and hugs, as well as the nature of the relationship whether casual acquaintances or close friends, may also impact microbial transmission. Research indicates that denser networks are associated with more significant microbial sharing, likely due to more frequent interpersonal contact facilitating microbial transfer. The design of these networks is equally influential. In centralized networks, where one individual holds a central position, this key player, or “network linchpin,” may experience more microbial exchanges due to their central role and extensive connections. Additionally, closer social ties within networks typically lead to greater microbial sharing, as closer relationships often involve more intimate interactions and shared environments.

### 3.3 Impact on Microbial Diversity

Social networks can affect the diversity of an individual's microbiome [59, 63]. Intimate interactions and shared object use within these networks can either enhance microbial diversity or alter the composition of the microbiome, with significant health implications. Preliminary studies suggest that socially active individuals, those with extensive social interactions and low social anxiety, tend to have more diverse skin microbiomes, likely due to the acquisition of new microbial species through human contact. However, microbial exchange in networks can also introduce potential pathogens, increasing health risks. Thus, the health effects of microbial sharing within networks are dual‐faceted.

### 3.4 Effects of Social Network Interventions

Interventions targeting social networks can lead to significant changes in individual microbiomes [17, 64]. For example, public health strategies that alter network dynamics can help prevent the spread of pathogens. During the COVID‐19 pandemic, measures such as face masks, remote working, and virtual gatherings proved effective against viral spread. Moreover, promoting healthy lifestyles and good hygiene practices, like reducing tobacco and alcohol consumption and maintaining oral hygiene, can improve an individual's microbial profile and reduce disease susceptibility. Future research should explore the potential benefits of such network interventions in modulating the microbiome.

In summary, social connections are crucial in shaping an individual's microbiome. As people navigate through social interactions, their microbiomes engage in a subtle yet significant “socio‐microbial dance.” Understanding microbial exchange patterns within social networks and their health implications enhances our grasp of the complex relationship between interpersonal dynamics and microbiomes. Future research should focus on how these networks influence microbiome stability and health, and investigate how strategic interventions in social networks can promote overall health improvement.

### 3.5 Mechanisms Underlying the Influence of Social Behavior on Microbial Dynamics

Social behavior encompasses meaningful interactions and activities occurring between individuals or groups. We broadly distinguish such behaviors by their psychosocial impact: supportive interactions, which include forms of emotional and material support, and stressful interactions, arising from conflict, exclusion, or socioeconomic hardship.

Having examined how social structures at both micro (family) and meso (social network) levels shape the human microbiome, we now explore the underlying mechanisms. This section employs a biopsychosocial framework to delineate the pathways linking social behavior to microbial dynamics. We systematically review biological, psychological and behavioral, and socio‐cultural environmental mechanisms. A clear understanding of these pathways will provide a theoretical basis for future interventions that target social factors to promote microbiome and public health.

### 3.6 Biological Pathways

Social interactions exert profound influences on microbial communities through diverse biological mechanisms [17, 36, 65, 66]. Primarily, physical intimacy serves as a direct conduit for the transfer of skin, oral, and gut microbes among individuals. This exchange, evident in various forms of closeness, significantly fosters microbial sharing within family units. The quality of social relationships, such as friendship or conflict, shows a significant association with levels of systemic immune biomarkers, including C‐reactive protein (CRP) [67]. Under stress, the brain further regulates the synthesis of peripheral immune mediators like IL‐18 through direct hormonal and neural signaling [68]. Fluctuations in these inflammatory factors can alter the gut microenvironment, thereby exerting a substantial influence on the human microbiome.

Moreover, social interactions can indirectly influence microbial compositions by impacting the immune system. Strong social connections and cohesive interpersonal relationships may mitigate stress and bolster immune functionality, which in turn can recalibrate microbial populations, promoting a harmonious balance of commensal and pathogenic microbes. The neuroendocrine pathway offers additional biological evidence. Studies indicate that strong social support can attenuate excessive activation in brain regions linked to social distress, including the dorsal anterior cingulate cortex (dACC) and Brodmann area (BA) 8 [69]. It also helps lower cortisol levels in response to social stressors. By modulating the hypothalamic‐pituitary‐adrenal (HPA) axis, these effects ultimately influence the intestinal environment and the balance of the gut microbiota [70].

### 3.7 Psychological and Behavioral Influences

Social interactions also shape microbial profiles through psychological and behavioral pathways [36, 71] and vice versa. Strong social networks and supportive relationships can improve mental well‐being, subsequently influencing lifestyle choices. Individuals within supportive social environments are more likely to engage in healthy behaviors, including balanced diets and strict hygiene practices, all of which favorably influence microbial landscapes. Emotional and social network support are positively associated with adherence to the Mediterranean diet. Adequate nutrition from such dietary patterns helps maintain the intestinal epithelial barrier, supports mucosal immunity, and supplies nutrients for microbial metabolism, thereby contributing to microbiome homeostasis [72, 73]. In contrast, inappropriate hygiene practices, such as improper handwashing or poor oral care, can disrupt the stability of local microbial communities and increase disease susceptibility [74, 75]. These examples illustrate how behavioral choices, shaped by social context, actively sculpt the human microbiome.

Additionally, social dynamics may indirectly shape microbial communities by modulating an individual's stress response. Stress is known to disrupt gut microbial equilibrium, a process potentially mediated by the bidirectional gut‐brain axis. Chronic stress, for example, can diminish beneficial bacterial populations such as Lactobacillus and elevate circulating levels of neuroactive metabolites like kynurenine, alterations linked to subsequent behavioral changes. Interventions that restore Lactobacillus abundance and rectify associated metabolic pathways can reverse these effects [76]. Thus, by enhancing stress resilience, social interactions could exert an indirect influence on the microbiome [66, 77].

On the contrary, it has also been shown that microbiome affects human behavior [66], as well as emotions and learning. However, the entire physiological processes (at the systems, cellular, and molecular levels) that mediate the contributions of microbes to host social behavior are not well understood. Due to the strong mechanistic and physical connections between the neurological and immune systems, behavioral abnormalities may result from any microbial effects on the immune system [78, 79]. As a result, it's possible that a large number of the effects reported in gut‐brain axis investigations actually represent an immunological response [80]. Further certain bacteria have the ability to inhibit the HPA axis' activation, demonstrating how the microbiome influences social behaviors by acting on distinct neural circuits in the brain that govern stress reaction [40]. Numerous signaling molecules involved in behavioral physiology, including glucocorticoids, amines, and neuropeptides, may be altered by the microbiome. Alternatively, their bioavailability can be regulated. Further studies are warranted to elucidate this complex interplay between microbe and social behavior.

### 3.8 Socio‐Cultural Environmental Factors

The expression of social behaviors within different socio‐cultural contexts can create distinct microbial community patterns [17, 81]. Dietary habits, lifestyle preferences, and hygiene standards, which vary widely across cultures, undoubtedly impact microbial configurations. Additionally, cultural norms related to physical intimacy and communal item usage can influence microbial transmission patterns. Socio‐cultural environments also vary in their stress levels and coping mechanisms. For example, individuals in high‐stress societies may experience heightened psychological stress, potentially destabilizing gut microbial balance. Conversely, societies with lower stress levels and stronger social support systems might promote microbial diversity and stability.

In summary, social behavior dynamically shapes an individual's microbial composition through a complex interactive network of biological, psychological, and socio‐cultural environmental factors. This process occurs not in isolation but via the integrated biological transduction of social signals, establishing a reciprocal link between the human microbiota and social conduct. The mechanisms encompass the direct physical transfer of microorganisms, the shaping of hygiene and dietary practices, and the bidirectional neuroendocrine‐immune communication mediated by the gut‐brain and HPA axes, along with direct neural signaling pathways. Future research should focus on these interactions, examining how different cultural and societal contexts affect microbial responses to social behaviors.

### 3.9 Health Implications of the Social Behavior‐Microbial Nexus

Social behavior has a significant impact on microbial communities through various mechanisms. Close interpersonal relationships and strong social support networks encourage lifestyles and mental states conducive to maintaining a balanced and diverse microbiome [64]. These benefits can have lasting positive effects on immune, digestive, and cognitive health [66]. However, social dynamics can also introduce negative changes to microbial ecosystems [65, 81]. Stressful social interactions can lead to psychological strain, disrupting gut microbiome balance. Additionally, social interactions can facilitate the spread of potential pathogens, increasing health risks [82]. Thus, the relationship between social behavior and microbial communities is inherently complex.

The extent and nature of health outcomes from this interaction depend on various factors, including genetic predispositions, environmental conditions, and lifestyle choices. For instance, strong social support networks can mitigate the negative effects of stress on gut microbiota. In contrast, strained social interactions might exacerbate psychological distress, disrupting gut microbiome balance and negatively impacting health.

## 4 Discussion

The interaction between social behavior and the microbiome is not isolated from structural socio‐political and contextual determinants. Factors such as urbanization, socioeconomic inequalities, public health policies, and environmental conditions significantly shape microbial diversity and resilience.

### 4.1 Urbanization and Industrialization

Urbanization has been linked to reduced microbiome diversity due to increased reliance on processed foods, exposure to environmental pollutants, and sedentary lifestyles [83]. Industrialization exacerbates these trends, as agricultural practices often rely on antibiotics and pesticides, which disrupt microbial ecosystems [84]. Multiple factors trigger physiological and psychological mechanisms that collectively reshape the microbial ecosystem. For example, urbanization reduces the diversity of commensal microbiota in humans. The abundance of specific microbial groups, such as Mycobacteria, Lactobacillus, and helminths decreases, particularly during early life. This affects the normal development and maturation of immune regulatory circuits, impairing the activity and function of regulatory immune cells, including regulatory T cells and regulatory macrophages, ultimately leading to immune dysregulation [85]. Such immune imbalance not only increases susceptibility to inflammatory diseases like allergies and autoimmune diseases, and mental disorders such as anxiety and depression, but may also further destabilize microbiome homeostasis through sustained inflammation.

Rural populations, in contrast, tend to have higher microbial diversity due to traditional dietary practices and closer interactions with natural environments. The “biogenics” hypothesis suggests that regular, intermittent exposure to a mixture of airborne bio‐compounds in natural environments inhibits the highly interconnected PI3K/Akt/mTORC1 signaling pathway [86]. This suppresses related pathological pathways and benefits physical and mental health. Therefore, future research should conduct comparative studies between urban and rural populations. It is necessary to deeply analyze the influencing factors and underlying mechanisms driving microbiome changes. This will help explore these dynamics within a broader social context and develop personalized preventive strategies for urban and rural residents.

### 4.2 Socioeconomic Inequalities

Disparities in income, education, and healthcare access particularly affect microbial health. Socioeconomic status during childhood, in particular, may have long‐term effects on gut microbiome composition. Studies have shown that lower parental education level and lower current socioeconomic status are associated with reduced abundance of Bacteroides species. Bacteroides is an important genus in the human gut microbiome, involved in regulating metabolic, immune, and barrier functions. This genus is closely associated with diseases such as Crohn's disease and alcoholic liver disease [87, 88].

Low socioeconomic status also influences behavioral patterns, which directly or indirectly affect microbial homeostasis. Marginalized populations often face food deserts, where access to fresh, microbiome‐supporting foods is limited. Instead, they rely on calorie‐dense, nutrient‐poor processed foods rich in saturated and trans fats, added sugars, and food additives. These dietary components directly alter microbiome function and metabolic processes [89]. In addition, individuals with low income often have limited health literacy and reduced access to healthcare, which hinders the establishment of a healthy microbiome. At the physiological and psychological level, these disparities are further compounded by higher stress levels, which reshape the microbiome through neuroendocrine pathways.

### 4.3 Public Health Policies and Structural Determinants

Policies governing sanitation, housing, and antibiotic stewardship play a critical role in shaping microbial ecosystems. For example, overcrowded housing increases the risk of pathogen transmission and reduces exposure to diverse environmental microbes, disrupting microbial homeostasis [90]. Conversely, greater urban green space promotes year‐round exposure to environmental microbiota, which benefits health [91]. Antibiotic overuse, often more prevalent in populations with unrestricted access or poor regulation, has been shown to deplete beneficial microbial populations and lead to antibiotic‐resistant strains [92]. In addition, unjust social policies and sudden economic crises can affect the physical and mental health of vulnerable populations [93], thereby activating chronic stress pathways and adversely affecting microbial homeostasis through the neuroendocrine‐immune axis.

Taken together, these structural factors influence microbial ecosystems primarily through three pathways: altering microbial exposure patterns, affecting human neuroimmune mechanisms, and reshaping microbial community structure. Therefore, considering microbiome regulation within the context of macro‐social policies and social structures may have broad and far‐reaching public health implications, ultimately benefiting larger populations.

### 4.4 Cultural Norms and Microbial Transmission

Cultural practices surrounding hygiene, food preparation, and physical contact influence microbial sharing and transmission patterns. In collectivist cultures, where communal dining and shared living spaces are more common, microbial exchange may be higher compared to individualistic societies. These cultural differences underscore the need for tailored public health interventions that consider both microbial health and cultural norms.

By recognizing the socio‐political and structural factors at play, we can better understand how these determinants mediate the relationship between social behavior and the microbiome. Addressing these factors through equitable policies and targeted interventions can mitigate negative impacts and promote microbiome health across populations.

### 4.5 Future Directions

This paper aims to elucidate the relationship between social behavior and microbial ecosystems and its health implications. Research indicates that social behavior affects an individual's microbial profile through biological, psychological, and socio‐cultural factors. The health implications of this relationship are influenced by genetic, environmental, and lifestyle factors.

However, existing research often fails to account for the macro‐social and structural contexts that shape both behavior and health, including socioeconomic inequalities and policy environments. Notable gaps include a lack of studies directly linking such structural factors to microbiome variation, an emphasis on environmental over social determinants, limited interdisciplinary frameworks, sparse integrated microbial‐social‐environmental data, and few longitudinal designs to track these dynamics.

Future research should integrate structural, socio‐political, and contextual factors to fully understand the social behavior‐microbiome relationship. Below are specific research directions based on these considerations:

1. Structural and Policy‐Level Studies. Research should investigate how socio‐political determinants, such as public health policies, urban planning, and food system regulation impact microbial diversity and health. For instance, studies could evaluate how policies promoting local, fresh food markets influence gut microbiome diversity in underserved communities.
2. Cross‐Cultural Comparisons. Examine how cultural norms and practices affect microbial transmission and diversity across different societies. For example, comparative studies between collectivist and individualistic cultures could illuminate how shared living spaces and communal dining influence microbiota.
3. Longitudinal Studies on Structural Shifts. Monitor microbial changes in populations undergoing structural shifts, such as urbanization or migration. Longitudinal studies would reveal how environmental, dietary, and social changes affect microbial ecosystems over time.
4. Interventions Addressing Inequities. Develop interventions targeting socioeconomic disparities in microbiome health, such as educational programs promoting nutritious diets, public health initiatives improving access to clean water and sanitation, and policies regulating antibiotic use. These interventions should also consider cultural appropriateness to maximize effectiveness.
5. Integration of Microbiome Monitoring in Public Health. Incorporate microbiome profiling into public health programs to identify populations at risk of dysbiosis and develop targeted strategies for prevention and treatment.

By addressing these structural and socio‐political factors, future research can offer more comprehensive insights into the relationship between social behavior and the microbiome. This integrative approach will facilitate the development of policies and interventions that improve both microbial and societal health, ultimately enhancing overall well‐being.

## Author Contributions

**Jing Li:** conceptualization, methodology, writing – original draft. **Yang Yang:** conceptualization, writing – original draft. **Yufeng Wang:** conceptualization. **Ji'An Liu:** writing – review and editing. **Xufeng Huang:** methodology, writing – review and editing. **Zhengrui Li:** conceptualization, writing – review and editing, methodology. **Ju Li:** conceptualization, writing – review and editing, methodology.

## Disclosure

The lead authors Zhengrui Li and Ju Li affirm that this manuscript is an honest, accurate, and transparent account of the study being reported; that no important aspects of the study have been omitted; and that any discrepancies from the study as planned (and, if relevant, registered) have been explained.

## Ethics Statement

The authors have nothing to report.

## Conflicts of Interest

The authors declare no conflicts of interest.

## Data Availability

The data that support the findings of this study are openly available in PubMed at https://pubmed.ncbi.nlm.nih.gov/.
